# Intelligent pressure-controlled percutaneous unroofing: advancing minimally invasive renal cyst treatment

**DOI:** 10.3389/fmed.2025.1579726

**Published:** 2025-04-30

**Authors:** Haijun Liao, Qiliang Zhai, Xin Huang, Chuance Du, Difu Fan, Yadong Li, Leming Song

**Affiliations:** ^1^Department of Nephrology, Ganzhou Hospital-Nanfang Hospital, Southern Medical University, Ganzhou, China; ^2^Department of Urology, Ganzhou Hospital-Nanfang Hospital, Southern Medical University, Ganzhou, China; ^3^Department of Urology, Nanfang Hospital, Southern Medical University, Guangzhou, China

**Keywords:** renal cyst, percutaneous unroofing, laparoscopic unroofing, minimally invasive surgery, pressure control

## Abstract

**Objective:**

Simple renal cysts (SRC) are common benign lesions that may require surgical intervention when symptomatic. This study aimed to compare the efficacy and safety of intelligent pressure-controlled percutaneous unroofing of renal cysts (IPC-PURC) with laparoscopic unroofing of renal cysts (LURC) in the treatment of SRC.

**Patients and methods:**

A retrospective analysis was conducted on 168 patients with SRC who underwent either IPC-PURC (*n* = 61) or LURC (*n* = 107) between December 2017 and December 2023. Key outcomes, including operative time, postoperative hospital stay, drainage duration, postoperative pain scores, hemoglobin decrease, and complication rates, were compared between the two groups.

**Results:**

The IPC-PURC group demonstrated significantly shorter operative times (78.3 ± 22.8 min vs. 108.6 ± 29.6 min, *p* < 0.001), postoperative hospital stays (4 days vs. 5 days, *p* < 0.001), and drainage tube durations (3 days vs. 4 days, *p* < 0.001) compared to the LURC group. Additionally, patients in the IPC-PURC group reported lower postoperative pain scores (*p* < 0.001). No significant differences were observed between the two groups in terms of hemoglobin decrease or complication rates. Both techniques achieved a 100% success rate in symptomatic relief and cyst resolution.

**Conclusion:**

IPC-PURC offers advantages in terms of shorter operative time, reduced postoperative hospital stay, and lower postoperative pain compared to LURC, while maintaining similar safety profiles and efficacy. Therefore, IPC-PURC may represent a superior minimally invasive option for the treatment of SRC.

## Introduction

Simple renal cysts (SRC) are common benign kidney lesions, with their prevalence increasing with age (1). While most renal cysts remain asymptomatic, some patients may develop complications such as flank pain, hypertension, or renal function impairment, necessitating surgical intervention (2). In recent decades, minimally invasive techniques have become the preferred treatment for symptomatic renal cysts, offering reduced trauma and quicker recovery compared to open surgery (3).

Laparoscopic unroofing of renal cysts (LURC) is an established minimally invasive procedure for treating SRC (4, 5). However, LURC has limitations, including the need for multiple port insertions, the potential for postoperative adhesions, and risks associated with pneumoperitoneum (6). To overcome these challenges and improve surgical outcomes, novel percutaneous techniques have been developed. Intelligent pressure-controlled percutaneous unroofing for renal cysts (IPC-PURC) is a new minimally invasive technique that employs real-time pressure monitoring and automated adjustment of irrigation fluid pressure within the cavity (7). This approach aims to maintain a stable operative field, reduce bleeding, and potentially improve the completeness of cyst wall removal, all while utilizing a single-access approach. Although initial experiences with IPC-PURC are promising, no comprehensive comparison with the conventional LURC technique has been reported.

This study aims to compare the clinical efficacy and safety of IPC-PURC and LURC in treating SRC. By analyzing key perioperative outcomes and complication rates, we seek to provide evidence-based insights that may guide clinical decision-making and improve the standard of care for patients with symptomatic renal cysts.

## Methods

### Study design and patient selection

This retrospective study was conducted at Ganzhou Hospital-Nanfang Hospital, Southern Medical University, between December 2017 and December 2023. The study protocol was approved by the institutional ethics committee, and informed consent was waived due to the retrospective nature of the study.

**Figure 1:**
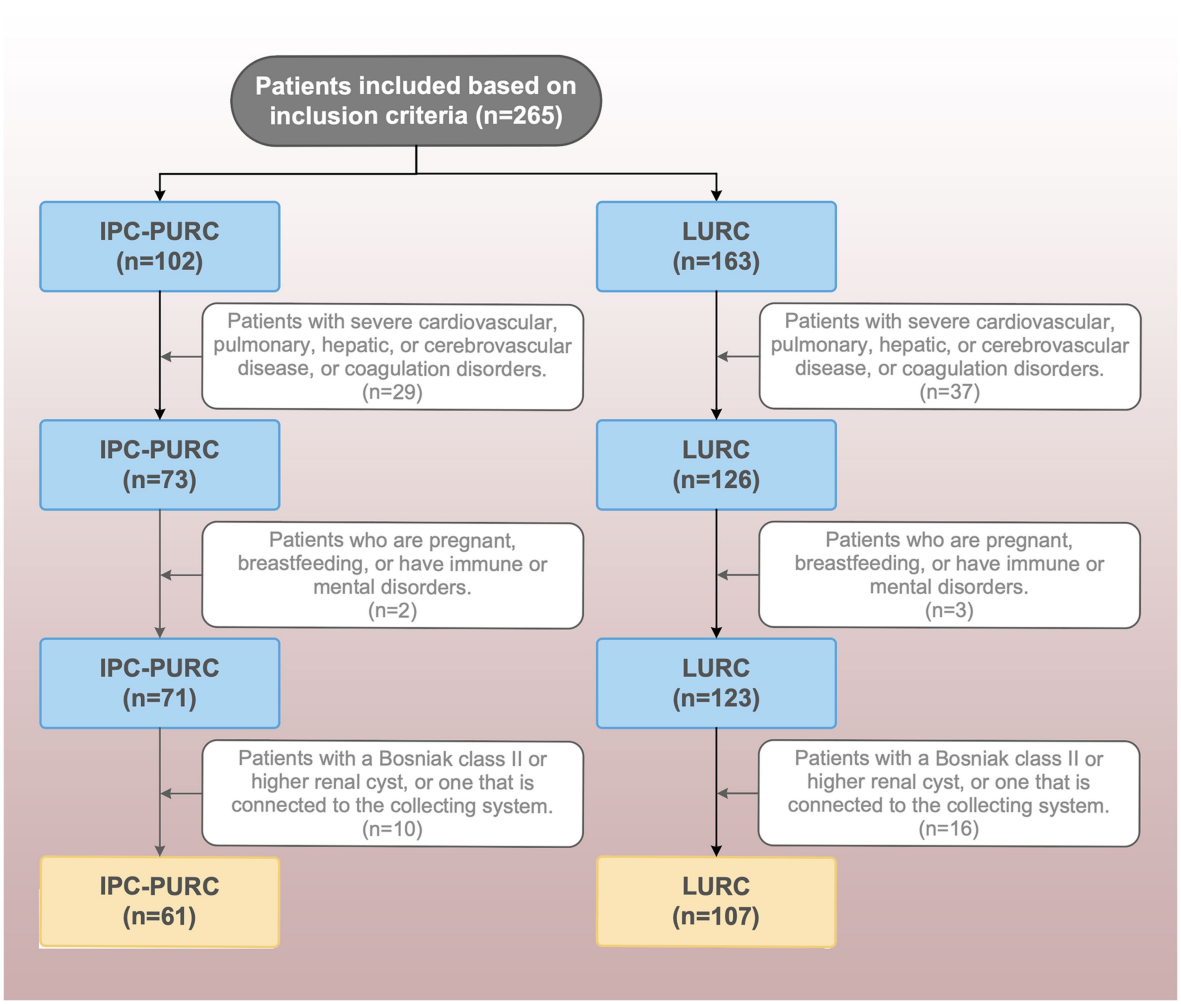
Flowchart of patient screening.

Data were extracted from our institution’s standardized research database (Digital Health China Technologies Co., Ltd., Beijing), which performs automated data desensitization, cleaning, and synchronization within 24 h post-discharge with weekly quality controls. Patient screening applied predefined inclusion/exclusion criteria through the platform’s embedded tools. As shown in Figure 1, inclusion criteria were: (1) age ≥18 years; (2) presence of a simple renal cyst confirmed by preoperative ultrasound and CT imaging; (3) cyst diameter ≥4 cm; (4) dorsally located exophytic renal cysts; and (5) Bosniak category I cysts; (6) patients with complete clinical records. Exclusion criteria included: complex or septated cysts, cysts associated with the collecting system, coagulation disorders, severe cardiopulmonary/hepatic/cerebrovascular disease, immune disorders, psychiatric conditions affecting compliance, and pregnancy or lactation.

Primary outcomes included operative time, postoperative hospital stay, drainage tube duration, postoperative pain scores (assessed using a visual analog scale [VAS] at 24 and 48 h post-surgery), and hemoglobin decrease. Secondary outcomes included intraoperative and postoperative complications, categorized according to the Clavien-Dindo classification system. According to the Clavien-Dindo classification system, grade I complications were defined as deviations from the normal postoperative course requiring pharmacological treatment, including but not limited to: (1) administration of analgesic medications for intractable pain (VAS ≥ 5) or pain-induced sleep disturbances; (2) antiemetics for persistent nausea/vomiting; (3) antipyretics for low-grade fever (≥ 37.3°C).

### Surgical techniques

#### IPC-PURC technique

The procedure was performed under general anesthesia with the patient in the prone position. Under ultrasound guidance, an 18-gauge needle was used to puncture the cyst. A guidewire was then inserted, followed by sequential dilation to accommodate an 18-Fr working sheath. The intelligent pressure-control system (Inventor Technology, Ganzhou, JiangXi, China) was connected to maintain a constant intracystic pressure of 10–15 mmHg throughout the procedure. A nephroscope was introduced for cyst wall inspection. The cyst wall was decorticated using a 1,470 nm laser fiber (600 μm, 100–120 W, 30–50 W) under direct vision. The pressure-control system automatically adjusted the irrigation flow to maintain a clear visual field and minimize bleeding. Detailed instrument specifications and procedural steps are provided in the Supplementary materials.

#### LURC technique

The procedure was performed under general anesthesia with the patient in the lateral decubitus position. Three ports were typically used: a 10-mm camera port and two 5-mm working ports. After establishing pneumoperitoneum, the cyst was identified and dissected free from surrounding structures. The cyst wall was excised, leaving a small rim attached to the renal parenchyma. The base of the cyst was fulgurated to prevent recurrence.

**Figure 2:**
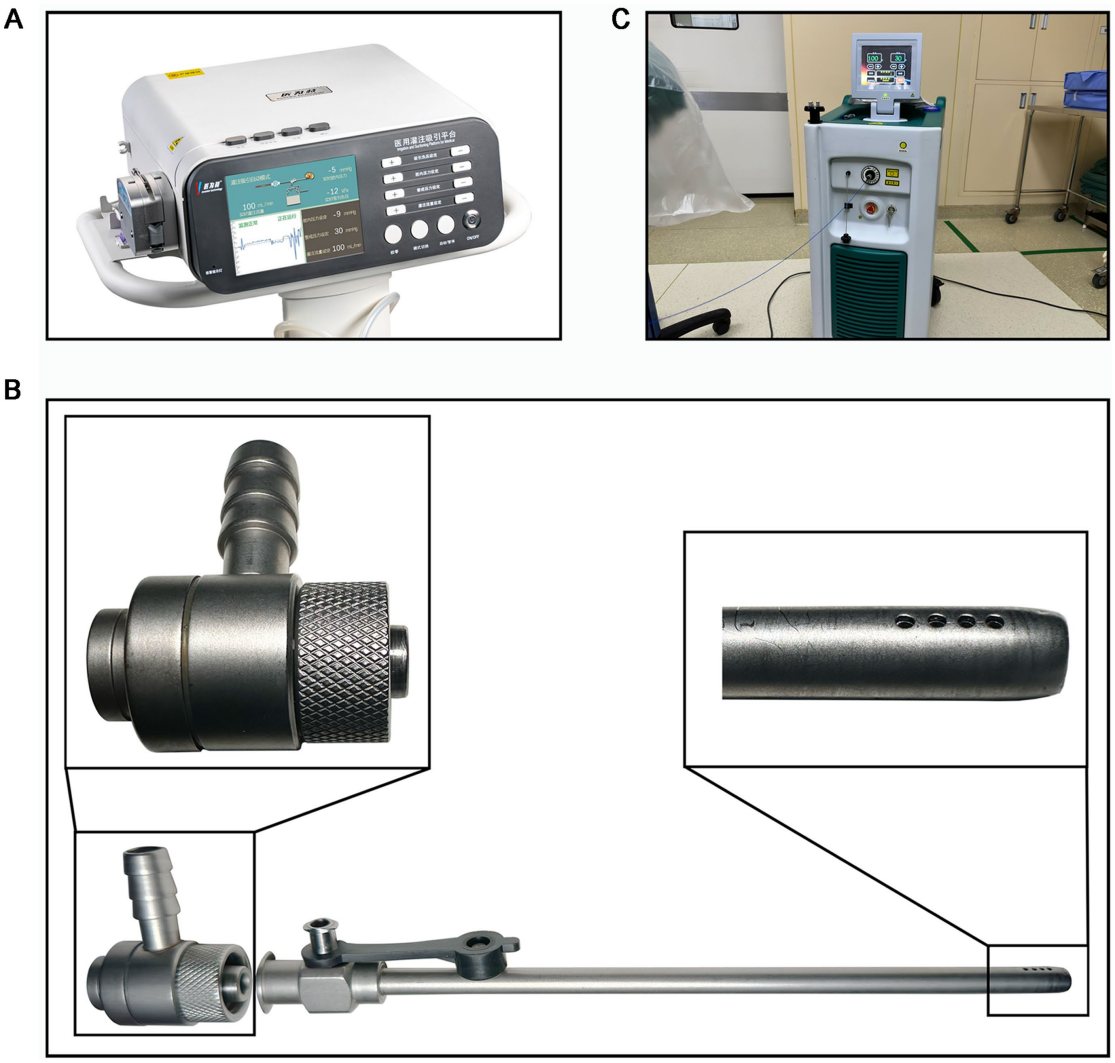
IPC-PURC and its supporting equipment **(A)** Intelligent air pressure system; **(B)** Intelligent pressure control sheath amplification detail; **(C)** 1,470 nm laser system.

In both groups, a drain was placed at the conclusion of the procedure. Figures 2A–C illustrates the key equipment.

### Statistical analysis

Continuous variables were expressed as mean ± standard deviation or median (interquartile range) depending on their distribution. Categorical variables were presented as frequencies and percentages. Between-group comparisons were performed using Student’s t-test or Mann–Whitney U test for continuous variables and chi-square or Fisher’s exact test for categorical variables. A *p*-value < 0.05 was considered statistically significant. All analyses were conducted using SPSS version 25.0 (IBM Corp., Armonk, NY, United States).

### Data availability

Data supporting the findings of this study are available from the corresponding author upon reasonable request.

## Results

### Demographic and clinical characteristics

A total of 168 patients were included in the study, with 61 patients in the IPC-PURC group and 107 patients in the LURC group (Table 1). There were no statistically significant differences between the two groups in terms of age, gender distribution, body mass index (BMI), preoperative hemoglobin levels, comorbidities, cyst laterality, cyst location, cyst size and follow-up period (all *p* > 0.05), indicating that the baseline characteristics were comparable.

**Table 1:** Baseline characteristics of patients in the IPC-PURC and LURC groups.

Variable	IPC-PURC	LURC	*P*-value
Number of patients	61	107	
Age, years, M (Q1, Q3)	58 (50.5, 65)	58 (51, 65)	0.715
Sex, *n* (%)			0.672
Male	31 (50.8%)	58 (54.2%)	
Female	30 (49.2%)	49 (45.8%)	
BMI	23.8 ± 2.7	24.4 ± 3.2	0.354
Preoperative hemoglobin, g/L	134.0 ± 16.2	134.7 ± 17.7	0.822
Postoperative hemoglobin, g/L	124.0 ± 18.2	122.5 ± 16.8	0.719
Comorbidity			
Hypertension	8 (13.1%)	19 (17.8%)	0.431
Diabetes	8 (13.1%)	12 (11.2%)	0.715
Coronary heart disease	3 (4.9%)	4 (3.7%)	1.000
COPD	6 (9.8%)	6 (5.6%)	0.306
Chronic kidney disease stage I-II	10 (16.4%)	13 (12.1%)	0.442
Cyst side, *n* (%)			0.272
Left	30 (49.2%)	62 (57.9%)	
Right	31 (50.8%)	45 (52.1%)	
Cyst location			0.834
Posterior upper	18 (29.5%)	27 (25.2%)	
Posterior middle	27 (44.3%)	50 (46.7%)	
Posterior lower	16 (26.2%)	30 (28.0%)	
Cyst size, cm, M (Q1, Q3)	6.5 (5.5, 7.6)	6.4 (5.5, 7.4)	0.708
Follow-up period, months, M (Q1, Q3)	6.0 (3.7, 8.1)	5.9 (3.5,8.3)	0.853

IPC-PURC, Intelligent pressure-controlled percutaneous unroofing for Renal Cyst; LURC, laparoscopic unroofing of renal cyst; M (Q1, Q3), Median (First quartile, Third quartile); BMI, body mass index; COPD, chronic obstructive pulmonary disease.

### Operative outcomes

The IPC-PURC group had significantly shorter operative times compared to the LURC group (78.3 ± 22.8 min vs. 108.6 ± 29.6 min, *p* < 0.001). Intraoperative blood loss was also significantly lower in the IPC-PURC group (2.8 ± 2.1 mL vs. 10.9 ± 3.5 mL, *p* < 0.001). Additionally, IPC-PURC patients experienced shorter postoperative hospital stays (median 4 days, IQR 3–6) compared to LURC patients (median 5 days, IQR 4–6) (*p* < 0.001) (Table 2).

**Table 2:** Comparison of operative outcomes between IPC-PURC and LURC groups.

Variable	IPC-PURC	LURC	*P*-value
Operative time, min	78.3 ± 22.8	108.6 ± 29.6	**<0.001**
Postoperative hospital stay, days, M (Q1, Q3)	4 (3, 6)	5 (4, 6)	**<0.001**
Efficacy, *n* (%)			1.000
Cured	61 (100%)	107 (100%)	
Invalid	0%	0%	
Drain tube time, days, M (Q1, Q3)	3 (2, 4)	4 (3, 5)	**<0.001**
Hemoglobin decrease, g/L	9.4 ± 7.3	11.7 ± 8.5	0.252
Postoperative VAS at 24 h			**<0.001**
0	37 (60.7%)	31 (29.0%)	
1–3	22 (36.1%)	65 (60.7%)	
4–6	2 (3.3%)	11 (10.3%)	
Postoperative VAS at 48 h			0.481
0	48 (78.7%)	79 (73.8%)	
1–3	13 (21.3%)	28 (26.2%)	
Clavien-Dindo classification grading system			
Grade I			
Pain^a^	9 (14.8%)	31 (29.0%)	**0.037**
Fever (≥ 37.3°C)	6 (9.8%)	8 (7.5%)	0.595
Nausea/vomiting	6 (9.8%)	11 (10.3%)	0.927
Grade II–V	0	0	

IPC-PURC, Intelligent pressure-controlled percutaneous unroofing for renal cyst; LURC, laparoscopic unroofing of renal cyst; M (Q1, Q3), Median (First quartile, Third quartile); VAS, Visual Analogue Scale (for pain assessment); Clavien-Dindo classification, A system used to grade the severity of surgical complications.

a Defined as patients requiring analgesic medication for pain relief.

### Postoperative recovery

The duration of drain tube placement was significantly shorter in the IPC-PURC group (median 3 days, IQR 2–4) compared to the LURC group (median 4 days, IQR 3–5) (*p* < 0.001). Although the decrease in hemoglobin levels was less in the IPC-PURC group, the difference was not statistically significant (9.4 ± 7.3 g/L vs. 11.7 ± 8.5 g/L, *p* = 0.252) (Table 2).

### Pain and complications

Postoperative pain scores at 24 h postoperatively were significantly lower in the IPC-PURC group (*p* < 0.001). In the IPC-PURC group, 60.7% of patients reported no pain (VAS score 0), compared to 29.0% in the LURC group. The incidence of moderate pain (VAS score 4–6) was also lower in the IPC-PURC group (3.3% vs. 10.3%) (Table 2). However, no significant differences were observed in pain scores between the two groups at 48 h postoperatively (*p* = 0.481).

Regarding Clavien-Dindo grade I complications, the IPC-PURC group had a significantly lower incidence of postoperative pain requiring additional analgesia (14.8% vs. 29.0%, *p* = 0.037). Other minor complications, such as fever and nausea/vomiting, were comparable between the two groups.

### Treatment efficacy

Both techniques achieved excellent efficacy, with a 100% success rate in terms of symptomatic relief and cyst resolution, as confirmed by follow-up imaging studies (Table 2). No cases of cyst recurrence were observed during the follow-up period (range: 3–24 months) in either group.

## Discussion

This study compared the efficacy and safety of IPC-PURC with LURC in the treatment of SRC. Our findings indicate that IPC-PURC offers significant advantages in operative time, postoperative hospital stay, drainage duration, and postoperative pain scores, while maintaining comparable safety and efficacy.

The IPC-PURC group demonstrated significantly shorter operative times (78.3 ± 22.8 min vs. 108.6 ± 29.6 min, *p* < 0.001), reduced postoperative hospital stays (4 days vs. 5 days, *p* < 0.001), and shorter drainage tube durations (3 days vs. 4 days, *p* < 0.001) compared to the LURC group. These improvements are likely due to several factors inherent in the IPC-PURC technique. The single-access approach eliminates the need for multiple port insertions and pneumoperitoneum, both of which are time-consuming steps in LURC (3, 6, 8, 9). Furthermore, the intelligent pressure control system in IPC-PURC maintains a stable operative field, potentially reducing the time required for hemostasis and enhancing overall surgical efficiency (7, 10). This underscores the potential of IPC-PURC to advance the field of minimally invasive renal cyst treatment.

The key innovation of IPC-PURC lies in its intelligent pressure control system, which offers several clinical benefits. By maintaining a constant intracystic pressure of 10–15 mmHg, the system ensures a clear visual field, reducing the risk of inadvertent injury to adjacent structures (7). The automated pressure regulation also likely contributed to the reduced intraoperative blood loss observed in the IPC-PURC group (2.8 ± 2.1 mL vs. 10.9 ± 3.5 mL, *p* < 0.001), as it helps to tamponade small vessels during cyst wall decortication. Moreover, the single-access approach minimizes surgical trauma and may reduce the risk of postoperative adhesions, a concern associated with LURC in previous studies (11–13). These factors likely contributed to the faster recovery and shorter hospital stays observed in the IPC-PURC group.

Both techniques demonstrated favorable safety profiles, with no significant differences in major complications (Clavien-Dindo grade II-V). However, the IPC-PURC group showed a significantly lower incidence of postoperative pain requiring additional analgesia (14.8% vs. 29.0%, *p* = 0.037). The reduced pain may be attributed to the less invasive nature of IPC-PURC, which avoids pneumoperitoneum and multiple port insertions (13). Lower postoperative pain scores in the IPC-PURC group (60.7% reporting no pain vs. 29.0% in LURC) further highlight the potential of this technique to enhance patient comfort and facilitate early mobilization, which could improve postoperative recovery and patient satisfaction.

Despite these advantages, IPC-PURC has limitations. The technique may be less suitable for anteriorly located or deeply situated renal cysts, which may be more easily accessed via a transperitoneal laparoscopic approach (4). Furthermore, variations in surgical billing standards and procedure-specific consumables preclude direct cost comparisons between techniques, which warrants exploration through standardized economic frameworks in future studies. Future advancements in technology, such as flexible operating instruments or enhanced imaging guidance, could broaden the applicability of IPC-PURC. Additionally, while this study focused on Bosniak I cysts, future research should explore the effectiveness of IPC-PURC in treating more complex cystic lesions. The pressure control system may require further refinement to manage septated or multilocular cysts effectively.

A major strength of this study is its relatively large sample size and well-matched baseline characteristics between the two groups, which enhance the reliability of our findings. However, as a single-center, retrospective study, the generalizability of our results may be limited. Although efforts were made to match patient characteristics, the potential for selection bias cannot be entirely ruled out. Furthermore, while our follow-up period was sufficient to assess short-term outcomes, longer-term follow-up would be beneficial for evaluating cyst recurrence and long-term renal function. Future prospective, multi-center randomized controlled trials are warranted to validate these findings and provide higher-level evidence.

The results of this study suggest that IPC-PURC could become the preferred minimally invasive technique for treating SRC, especially in patients where rapid recovery is paramount. The shorter operative times and hospital stays associated with IPC-PURC could improve resource utilization and cost-effectiveness, which warrants further investigation. Future research should also focus on long-term outcomes, including cyst recurrence rates and the potential impact on renal function over time (14–16). Additionally, studies exploring the learning curve of IPC-PURC and its applicability to more complex renal cysts (e.g., Bosniak II cysts) would be valuable. Investigating the combination of IPC-PURC with other minimally invasive techniques, such as laser ablation of the cyst wall, could also be an interesting avenue for future research.

In conclusion, this study demonstrates that intelligent pressure-controlled percutaneous unroofing of renal cysts (IPC-PURC) offers significant advantages over laparoscopic unroofing of renal cysts (LURC) in terms of operative efficiency, postoperative recovery, and patient comfort, while maintaining comparable safety and efficacy.

## Data Availability

The raw data supporting the conclusions of this article will be made available by the authors, without undue reservation.
