# Supplementary material for: Intelligent pressure-controlled percutaneous unroofing: advancing minimally invasive renal cyst treatment
Source: Front Med (Lausanne). 2025 Apr 30;12:1579726. doi: 10.3389/fmed.2025.1579726 (PMC12075117; doi:10.3389/fmed.2025.1579726)
Supplement: Supplementary file 1 [file Data_Sheet_1.pdf]

Supplementary Material

Intelligent Pressure-Controlled Percutaneous Unroofing: Advancing Minimally Invasive Renal Cyst Treatment

Intelligent pressure-controlled (IPC) system

Instructions for use

1.1 Platform startup and working mode

Connect the detachable power cord in the chassis to the platform host and plug it into a power socket with a ground wire (protective ground). Turn the platform host power switch to the ‘I’ position, then press the host’s “ON/OFF” switch to turn on the platform power.

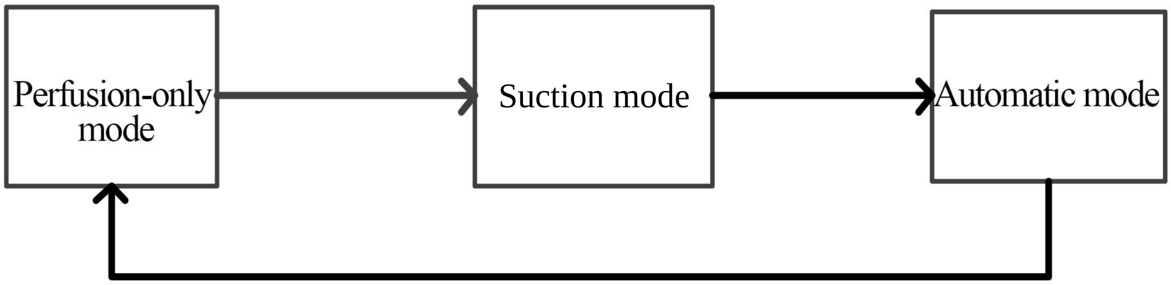

Figure 1-1 Mode switching process

After startup, the system defaults to the perfusion-only mode, which can be used normally. The other two modes are entered by pressing the ‘Mode/Switch’ key. The process is shown in Figure 1-1.

1.2 Working mode self-check

a. Physiological perfusion mode does not require self-check.

b. Self-check of suction mode.

If the interface shown in Figure 1-2 is displayed, a self-check is required. Perform the self-check according to the requirements of the self-check interface. After the self-check is successful, it can be used normally. (Remove the vacuum terminal connector during self-check, and recover after successful self-check).

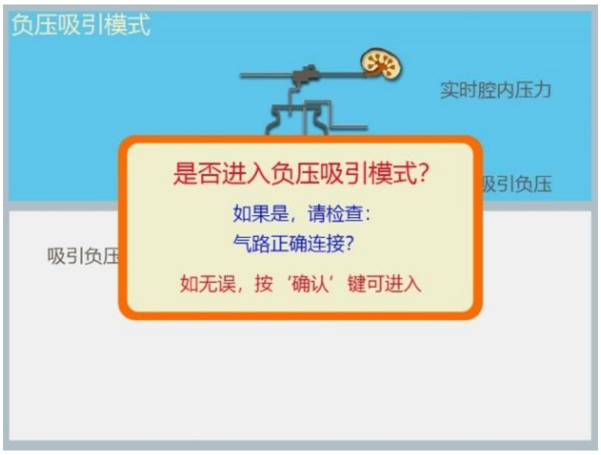

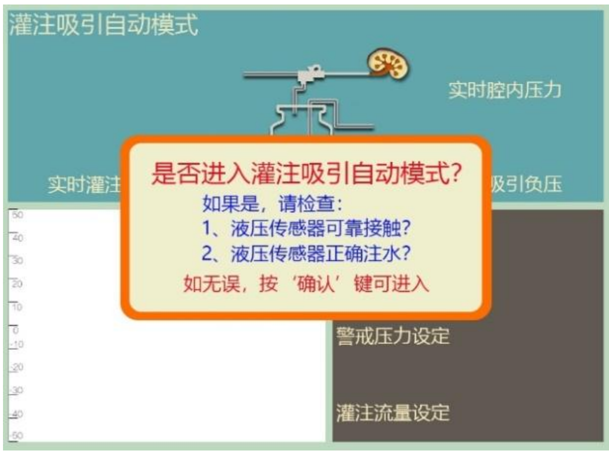

Figure 1-2 Self-check interface of suction mode

Figure 1-3 Self-check interface of automatic mode

c. Automatic mode self-check.

If the interface shown in Figure 1-3 pops up, perform self-test according to the interface requirements. After successful self-test, it will exit the self-test interface and can be used normally. After properly placing the endoscopic introducer or disposable pressure-measuring ureteral access sheath into the renal pelvis, use a 20ml syringe to inject saline to evacuate the pressure measurement tube. It can be seen that the liquid at the end of the endoscope introducer or disposable pressure-measuring ureteral access sheath is flowing out. And at the same time, press the "OK" button to complete the self-check.

Note: After each start-up, the platform needs to perform a self-check once before entering the automatic mode or suction mode. The self-tests in the two modes are independent of each other.

1. 3 Working mode interface

After the self-check is completed, press the ‘Mode/Switch’ key to switch the system to different working modes, which are divided into automatic mode, physiological perfusion-only mode and suction mode. The corresponding mode name is displayed in the screen, as shown in Figure 1-4, Figure 1-5, and Figure 1-6.

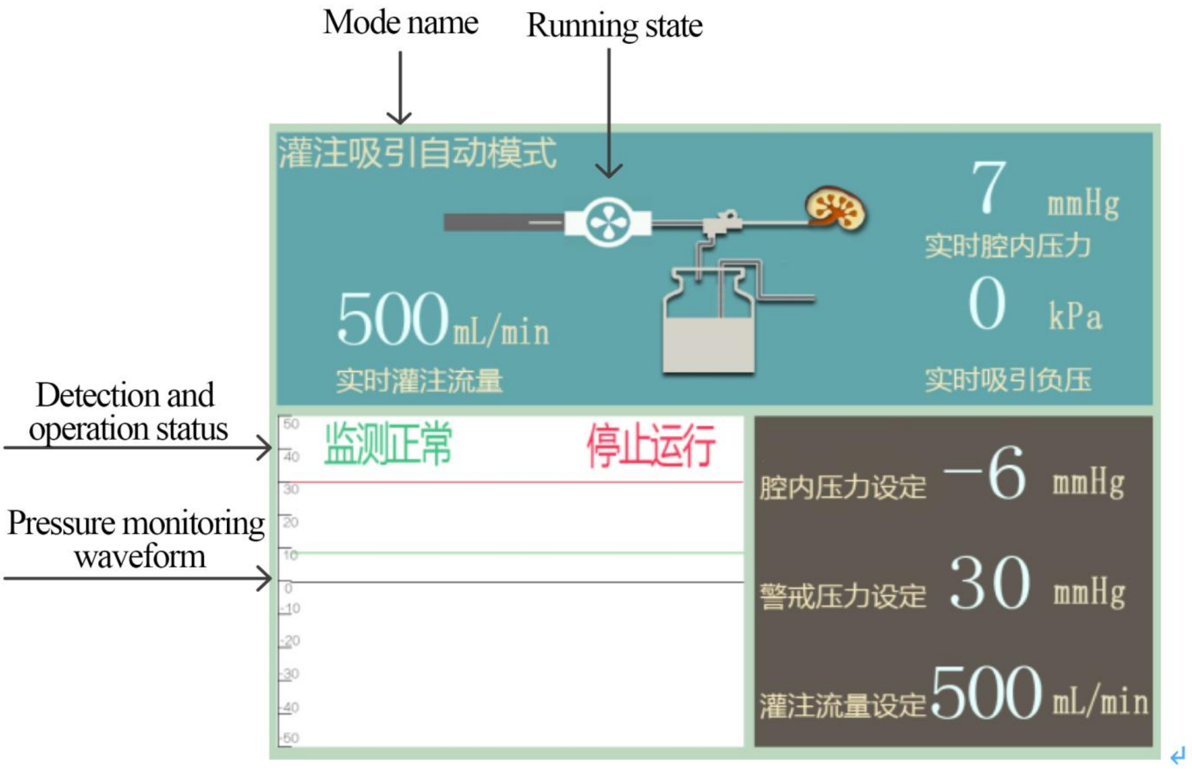

Figure 1-4 Interface of automatic mode.

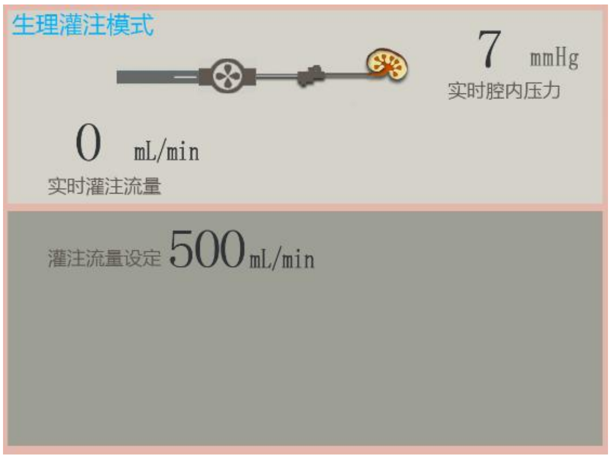

Figure 1-5 Perfusion mode interface

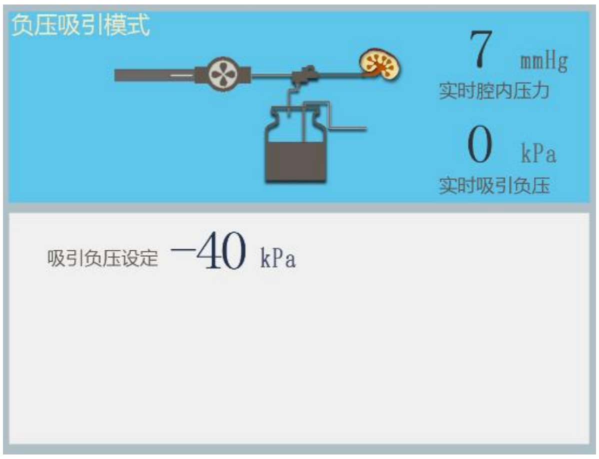

Figure 1-6 Suction mode interface

1. 4 Description of working mode

1. 4. 1 Perfusion-only mode

In perfusion-only mode, the peristaltic pump and hydraulic sensor are in working state. The peristaltic pump is automatically filled according to the set value of the perfusion flow, and the

perfusion flow is displayed on the screen. In addition, the platform displays the detected pressure on the screen in real time through a hydraulic sensor. This mode has no alarm function.

1.4.2 Suction mode

In the suction mode, the vacuum pump and hydraulic sensor are in working state. The platform monitors the negative pressure in the collection container in real time. When the real-time suction negative pressure is greater than the suction negative pressure setting value, the negative pressure suction will stop and the detected negative pressure value will be displayed on the screen in real time. In addition, the platform displays the detected pressure value on the screen in real time through a hydraulic sensor. This mode has no alarm function.

1.4.3 Automatic mode of perfusion and suction

In the automatic mode, the peristaltic pump, vacuum pump, hydraulic sensor and alarm system are in working state. When this mode is activated, the peristaltic pump fills the saline into the cavity according to the set value of the perfusion flow, and the hydraulic sensor monitors the liquid pressure value in the cavity in real time. The real-time perfusion flow and the real-time intracavity pressure are displayed on the screen. The platform automatically adjusts the vacuum pump to form a negative pressure according to the monitored pressure value of the liquid in the cavity, and sucks the liquid out through the endoscope introducer or disposable pressure-measuring ureteral access sheath. Thereby maintaining a safe range of liquid pressure in the cavity. In addition, the platform will monitor the negative pressure in the collection container in real time and display the negative pressure value on the screen in real time.

Set the liquid pressure reference zero point in the cavity. After the self-check is completed and the real-time pressure value is relatively stable. Press the 'Zero Calibration' button. The zero-calibration interface shown in Figure 3-7 will pop up. Press the 'Confirm' button to exit the zero-calibration interface. The intracavity pressure display on the screen is 0 mmHg. At this time, the pressure in the kidney is set to 0 mmHg, which means that the zero-calibration is successful. If the ‘confirmation’ key is not pressed within the valid time, the zero calibration is

invalid this time and the zero-calibration interface is automatically exited. If you press the ‘Zero’ button again after successful zero calibration, it will be deemed invalid by the platform and a warning interface will appear on the screen for 2 seconds, as shown in Figure 3-8. If you need to zero again, you can only restart the platform.

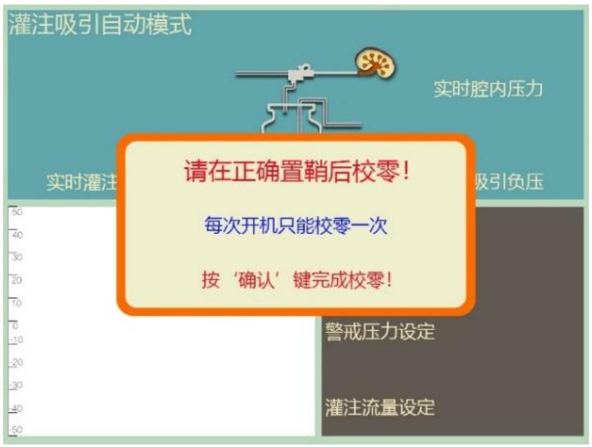

Figure 1-7 Zero-calibration operation interface

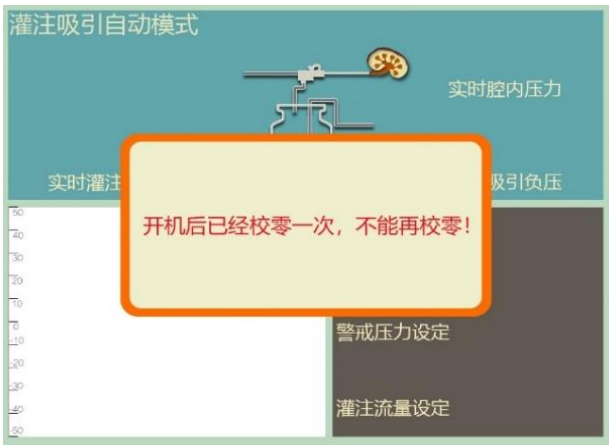

Figure 1-8 Repeat zero-calibration warning interface

Note: To ensure that the relative pressure in the cavity is accurate during the operation, you can only perform zero calibration once after each boot. In physiological perfusion mode and negative pressure suction mode, there is no zero calibration function.

1.5 Parameter setting

1.5.1 Perfusion flow setting

In automatic mode and perfusion-only mode, set the perfusion flow value by adjusting the '+' button or '-' button of the 'perfusion flow setting' on the front panel. And the set value displays 'Perfusion flow setting XXX mL/min" on the screen, and the setting range is 50～500mL/min.

1.5.2 Suction negative pressure setting

In the suction mode, set the suction negative pressure value by adjusting the '+' button or '-' button on the front panel 'Suction negative pressure setting'. And the set value displays ' Suction negative pressure setting XXX kPa' on the screen, the setting range is 0～-50kPa.

1.5.3 Intracavity pressure setting

In the automatic mode, set the intracavity pressure by adjusting the '+' button or '-' button in the 'Intracavity pressure setting' on the front panel. The set value displays ' Intracavity pressure setting XXX mmHg' on the screen, the setting range is -20～20mmHg.

Note: It is recommended to set the intracavity pressure between -15mmHg～-2mmHg.

1.5.4 Alarm pressure setting

In the automatic mode, set the warning pressure value by adjusting the '+' button or '-' button in the 'Alarm pressure setting' on the front panel (default is 30mmHg) to ensure the safety of the surgical process. The set value displays 'Alarm pressure setting XXX mmHg' on the screen, and the setting range is 0～40mmHg.

Note: In PCNL, there is a height (cmH2O, 1mmHg=1.36cmH2O) from the end of the endoscope introducer to the kidney. So this height difference needs to be reduced when setting the alarm pressure. Alarm pressure setting value = 30mmHg - height difference (mmHg).

1.6 Data storage

1.6.1 Data copy

In the paused state, insert the USB divice into the USB port on the back of the platform. Press the record button above the platform, the storage confirmation interface shown in Figure 3-9 will pop up. Press the record button again within 10s, The storage progress display interface shown in Figure 3-10 will appear. After loading to 100%, the data has been successfully stored, otherwise the storage fails as shown in Figure 1-11. (The other modes will also prompt the same)

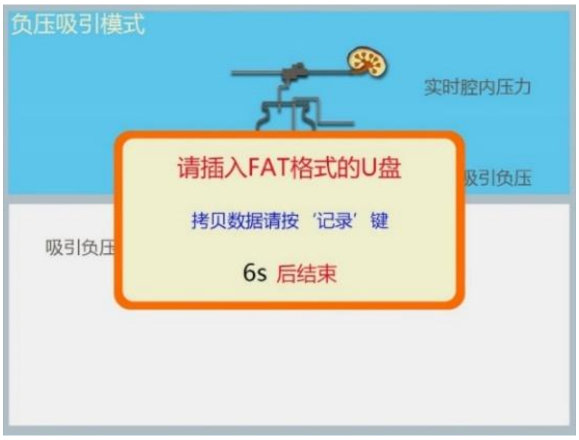

Figure 1-9 Storage confirmation interface

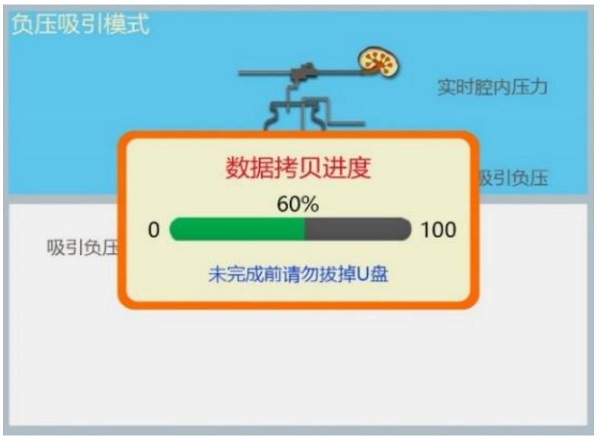

Figure 1-10 Storage progress display interface

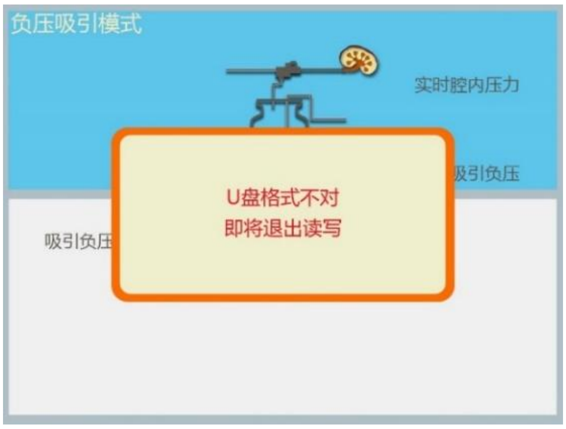

Figure 1-11 Storage error interface

Note 1: Do not remove the USB divice during data copying, otherwise data copying will fail;
Note 2: Only data within three days can be copied.

1. 6. 2 Data format

a. The system creates a folder directory in the format of year, month, and day. The first-level directory is displayed in years. After entering the year folder, it is displayed in months. After entering the month folder, it is displayed in days. After entering the day folder, the data table of each boot record is displayed. The data table suffix is in CSV format, which can be opened and viewed using Excel. As shown in Figure 3-12 below, KINGSTON (D:) is the name of the U disk, the data table is stored under the 2019 folder-May folder-5th folder. The CSV files are

data files that be created separately at each startup.

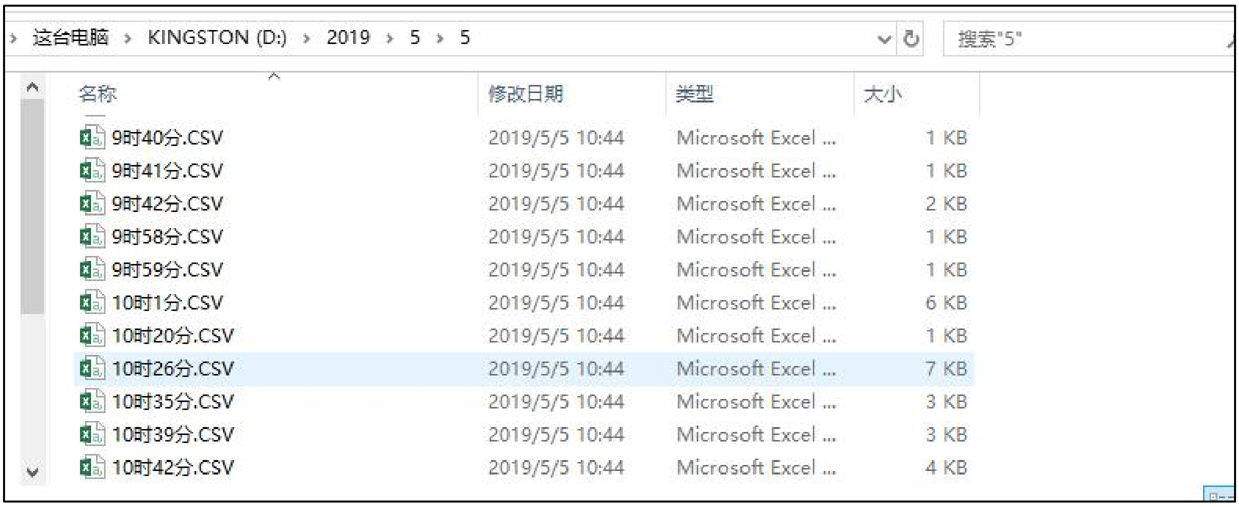

› 这台电脑 › KINGSTON (D:) › 2019 › 5 › 5   搜索"5"

名称	修改日期	类型	大小
9时40分.CSV	2019/5/5 10:44	Microsoft Excel ...	1 KB
9时41分.CSV	2019/5/5 10:44	Microsoft Excel ...	1 KB
9时42分.CSV	2019/5/5 10:44	Microsoft Excel ...	2 KB
9时58分.CSV	2019/5/5 10:44	Microsoft Excel ...	1 KB
9时59分.CSV	2019/5/5 10:44	Microsoft Excel ...	1 KB
10时1分.CSV	2019/5/5 10:44	Microsoft Excel ...	6 KB
10时20分.CSV	2019/5/5 10:44	Microsoft Excel ...	1 KB
10时26分.CSV	2019/5/5 10:44	Microsoft Excel ...	7 KB
10时35分.CSV	2019/5/5 10:44	Microsoft Excel ...	3 KB
10时39分.CSV	2019/5/5 10:44	Microsoft Excel ...	3 KB
10时42分.CSV	2019/5/5 10:44	Microsoft Excel ...	4 KB

Figure 1-12 Data directory format

b. Open the CSV file, you can view the system start time recorded in the data table, real-time intracavity pressure, real-time suction negative pressure, intracavity alarm pressure setting, intracavity pressure setting, perfusion flow, system mode and system status at different time points (Run 1, stop 0), as shown in Figure 1-13 below. (System mode A: Automatic mode; System mode B: Perfusion-only mode; System mode C: Suction mode.)

	A	B	C	D	E	F	G	H
1	系统开启时间:	2019年5月5日 10:42:32						
2	时间	实时腔内压力	实时吸引负压	腔内警戒设定	腔内目标设定	灌注流量	系统模式	系统状态（运行1，停止0）
42	10:43:12	1	0	30	-9	110	A	1
43	10:43:13	2	0	30	-9	110	A	1
44	10:43:14	3	0	30	-9	110	A	1
45	10:43:15	2	-1	30	-9	110	A	1
46	10:43:17	2	0	30	-9	110	A	1
47	10:43:18	0	-1	30	-9	110	A	1
48	10:43:19	-14	0	30	-9	110	A	1
49	10:43:19	-17	0	30	-9	110	A	1
50	10:43:20	-16	0	30	-9	110	A	1
51	10:43:24	-13	0	30	-9	110	A	1
52	10:43:24	-11	0	30	-9	110	A	1
53	10:43:25	-10	0	30	-9	110	A	1
54	10:43:25	-8	0	30	-9	110	A	1
55	10:43:26	-7	0	30	-9	110	A	1
56	10:43:27	-7	0	30	-9	110	A	0
57	10:43:27	-6	0	30	-9	110	A	0
58	10:43:28	-5	0	30	-9	110	B	0
59	10:43:28	-6	0	30	-9	110	B	0
60	10:43:29	-5	0	30	-9	110	B	0
61	10:43:29	-6	0	30	-9	112	B	0
62	10:43:30	-5	0	30	-9	114	B	0
63	10:43:30	-5	0	30	-9	115	B	0
64	10:43:31	-5	0	30	-9	116	B	0
65	10:43:31	-5	0	30	-9	119	B	0
66	10:43:32	-5	0	30	-9	120	B	0
67	10:43:32	-5	0	30	-9	120	B	1
68	10:43:34	-5	0	30	-9	120	B	0
69	10:43:37	-5	0	30	-9	120	A	0
70	10:43:38	-5	0	30	-9	120	B	0
71	10:43:40	-5	0	30	-9	120	C	0

Figure 1-13 Contents of the data table

1.7 Operating instructions for collecting containers

During the operation, the user should replace the collection container in time before the collection container is full to prevent the liquid from entering the platform host.

Replacement process:

a. Inform the surgeon in advance;

b. Press the "Start/Pause" button to stop the platform and the endoscope exits the sheath;

c. Replace the collection container (usually two collection bottles and one cap, and replace the cap of the collection container filled with liquid with an empty collection container);

d. After replacing, press “Start/Pause” button to continue the operation.

Alarm System

1.8 Check the alarm system

In automatic mode, change the pressure source of the hydraulic sensor (disposable pressure-measuring ureteral access sheath) to make the platform send out an audible and visual alarm signal when the real-time intracavity pressure is higher than the set value of the alarm pressure.

1.9 Alarm system status

The alarm status of the medical perfusion and suction platform is divided into low, medium and high priority. The alarm signal is prompted by the alarm indicator (optical signal) and the speaker (acoustic signal).

a. Low priority alarm: In the automatic mode, when the value of ‘alarm pressure setting’ is higher than 30mmHg, it is defined as a low priority alarm state. At this time, the alarm indicator light is continuously on in yellow, and the speaker emits a sound alarm, and the platform can continue to work.

b. Medium priority alarm: In the automatic mode, when the value of 'intracavity pressure' rises to 5mmHg different from the value of 'alarm pressure setting', it is defined as the medium priority alarm state. At this time, the alarm indicator flashes yellow, the speaker sounds an alarm, and the platform can continue to work.

c. High-priority alarm: In the automatic mode, when the value of ‘intracavity pressure’ exceeds the value of ‘alarm pressure setting’, it is defined as a high-priority alarm state. At this time, the alarm indicator flashes red, the speaker emits a sound alarm, and the platform stops working.

In any alarm state, press the ‘alarm pause’ key to clear the alarm (press the ‘mute’ key to eliminate the sound only). Regardless of whether the alarm is eliminated or not, when the value of ' intracavity pressure' returns to below the value of 'Alarm pressure setting' (default is 30mmHg). Press "Start/Pause" key, the platform will start the automatic mode, and the alarm signal is automatically eliminated.

1.10 Alarm signal standard

The visual and audible alarm signals comply with the requirements in Table 202, Table 203 and Table 204 of YY 0709-2009.

1.11 Alarm system delay

The hydraulic sensor continuously collects the liquid pressure in the cavity. After the collected data is

converted by analog-to-digital (A/D), it is transferred to the CPU for the average value of the liquid pressure. The CPU makes a logical judgment based on the average value of the liquid pressure and the value of the 'alarm pressure setting' value. For example: when the average value of the liquid pressure is greater than the value of ‘alarm pressure setting’, an audible and visual alarm signal is issued, and the operation of the vacuum pump and the peristaltic pump is stopped at the same time. The delay time of the alarm system = A/D conversion time + CPU averaging time + turn off the negative pressure pump / peristaltic pump time. The A/D conversion time is 43ms, the time for the CPU to average is 21.5us, and the time to turn off the negative pressure pump/peristaltic pump is 510ms (the longest time to turn off). That is, the maximum delay time of the alarm system is about 553.0215ms, which is less than 1s.

1. 12 Alarm preset

The alarm preset set by the manufacturer is: The ‘alarm pressure setting’ is 30mmHg. The user can restore the preset value by long pressing the ‘+’ button on the front panel for more than 3s. When the power failure time does not exceed 30s, the alarm setting before power failure is automatically restored.

1. 13 Alarm limit

After pressing the "Zero-calibration" key, the operator is allowed to set the value of "Alert Pressure Setting" to the limit value, the limit value is 40mmHg. When the operator sets (or adjusts) the alarm limit, the alarm system will continue the normal function with the new limit.
